# Comparison of Catheter Ablation in Patients with Paroxysmal Non-valvular Atrial Fibrillation and Heart Failure with Preserved Ejection Fraction

**DOI:** 10.7150/ijms.103170

**Published:** 2025-01-01

**Authors:** Zixi Zhang, Qiuzhen Lin, Cancan Wang, Keke Wu, Yunying Huang, Tao Tu, Zeying Zhang, Hanze Tang, Yichao Xiao, Qiming Liu

**Affiliations:** 1 Department of Cardiology, The Second Xiangya Hospital, Central South University, Changsha 410011, Hunan Province, People's Republic of China.; 2 Department of Endocrinology, The Second Xiangya Hospital, Central South University, Changsha 410011, Hunan Province, People's Republic of China.

**Keywords:** Atrial fibrillation (AF), Heart failure with preserved ejection fraction (HFpEF), Radiofrequency ablation (RFA), Cryoballoon ablation (CBA), Pulsed field ablation (PFA).

## Abstract

**Background:** The efficacy of radiofrequency ablation (RFA) in patients with atrial fibrillation (AF) and heart failure with preserved ejection fraction (HFpEF) has been established, but the efficacy and safety of cryoballoon ablation (CBA) and pulsed field ablation (PFA) remain unclear.

**Methods:** This retrospective cohort study included 223 patients with paroxysmal non-valvular AF and HFpEF who underwent their first AF ablation between January 2017 and December 2021 and were divided into RFA (n = 77), CBA (n = 127), and PFA (n = 19) groups.

**Results:** After a mean follow-up of 11.2 ± 1.8 months, no significant differences were observed in the rates of AF recurrence among the groups (*P* = 0.964). Both RFA and PFA were associated with a reduction in left atrial diameter and an increase in left ventricular ejection fraction (LVEF), whereas CBA showed no significant changes. The New York Heart Association (NYHA) functional classification and quality of life scores significantly improved across all groups (*P* < 0.01). No significant differences in the incidence of postprocedural complications were observed. Multivariate Cox regression analysis identified serum albumin (ALB) and N-terminal pro-B-type natriuretic peptide (NT-pro BNP) as independent predictors of AF recurrence post-ablation.

**Conclusion:** RFA, CBA, and PFA are all effective in maintaining sinus rhythm in patients with paroxysmal non-valvular AF and HFpEF. RFA and PFA were associated with improved quality of life, improved NYHA functional classification, reversal of atrial remodeling, and increased LVEF. While CBA improved quality of life and NYHA functional status, it did not reverse atrial remodeling or increase LVEF. ALB and NT-pro BNP levels were identified as independent predictors of AF recurrence post-ablation in HFpEF patients.

## Introduction

Atrial fibrillation (AF) is the most common type of sustained arrhythmia, with an estimated lifetime risk of approximately 30% in adults 1. AF is associated with a significantly increased risk of adverse outcomes, including a 2.4-fold greater risk of stroke and a 10.5-fold greater risk of heart failure (HF) 2. Heart failure with preserved ejection fraction (HFpEF), a major subtype of HF, is characterized by intricate bidirectional interactions with AF 3, representing a substantial public health burden.

Conventional ablation therapies, such as radiofrequency ablation (RFA) and cryoballoon ablation (CBA), have become essential strategies in the management of AF. An in-depth analysis of the CABANA trial data (trial code NCT00911508) by Packer *et al.* 4 demonstrated that RFA outperformed antiarrhythmic drugs in improving quality of life and reducing AF recurrence in patients with HFpEF. Furthermore, a meta-analysis indicated that RFA for AF in patients with HFpEF was as safe and effective as RFA for maintaining sinus rhythm in patients with heart failure with reduced ejection fraction (HFrEF) 5. A previous study indicated that CBA can alleviate HF symptoms, reduce hospitalization time, and reverse remodeling in patients with AF and HFpEF 6. However, recent research suggests that CBA may not improve quality of life or diastolic function in this cohort and could even increase the risk of AF recurrence after ablation 7. These findings present a "therapeutic paradox", raising concerns about the long-term efficacy and safety of CBA in patients with AF and HFpEF. As a result, RFA may offer a more effective treatment option than CBA does, although further evidence is needed for confirmation.

Pulsed field ablation (PFA) is an emerging ablation technology that uses high-frequency pulsed electric fields to create micropores in cell membranes. This method offers the potential for safe and efficient ablation while preserving surrounding vascular and neural tissues 8. While the efficacy and safety of PFA in patients with AF and HFpEF have yet to be fully validated, its ability to achieve efficient pulmonary vein isolation (PVI) makes it a promising candidate for rhythm control in the future.

Given the current lack of robust evidence on the efficacy and safety of CBA and PFA in treating patients with AF and HFpEF, this study aimed to evaluate electrophysiological remodeling, structural changes, and functional outcomes in patients with paroxysmal non-valvular AF and HFpEF following ablation. This assessment will assist clinicians in evaluating these patients prior to AF ablation and in developing personalized treatment strategies. Furthermore, this study aimed to identify predictive factors for AF recurrence in this population, facilitating early interventions that could reduce recurrence rates and improve long-term prognosis.

## Methods

### Study design and participant selection criteria

This was a retrospective, observational cohort study conducted at the Cardiology Department of the Second Xiangya Hospital. The study retrospectively screened all patients aged over 18 years who were diagnosed with AF and who underwent catheter ablation (including RFA, CBA, and PFA) between January 1, 2017, and December 31, 2021. Patients who did not meet the inclusion criteria were excluded. The final cohort consisted of individuals with paroxysmal non-valvular AF and HFpEF, who were categorized into RFA, CBA, and PFA groups on the basis of the type of ablation energy used. The study evaluated various outcomes, including AF recurrence, atrial remodeling, changes in left ventricular ejection fraction (LVEF), quality of life scores, New York Heart Association (NYHA) functional classification, and postprocedural complications. The primary endpoint of this study was a composite of mortality and AF recurrence. The secondary endpoints included: (1) repeat AF ablation, AF-related rehospitalizations, AF-related emergency department visits, AF-related cardioversion, resumption of antiarrhythmic drug therapy at follow-up, and other arrhythmia-related events; (2) deterioration of cardiac function; and (3) the occurrence of procedure-related complications.

Eligibility for inclusion required all patients to have had at least one documented episode of AF, identified by a 12-lead electrocardiogram (ECG) or a single-lead ECG lasting more than 30 seconds. HFpEF was diagnosed according to the 2021 diagnostic criteria of the European Society of Cardiology 9, which include clinical signs or symptoms of HF (NYHA Class II-IV), preserved LVEF (≥ 50%), elevated levels of N-terminal pro-B-type natriuretic peptide (NT-pro BNP) (> 125 pg/mL), and evidence of diastolic dysfunction on structural and/or functional echocardiography. Additionally, the diagnosis of HFpEF was validated via the H2FPEF score, as recommended by the 2023 American College of Cardiology Expert Consensus Decision Pathway 10. All patients diagnosed with HFpEF had H2FPEF scores > 5 points. The exclusion criteria included patients with acute decompensated HF or cardiogenic shock, severe valvular heart disease, acute myocardial infarction, hepatic or renal dysfunction, malignancy, a left atrial diameter (LAD) > 50 mm, or pregnancy. Patients underwent additional linear ablation beyond the PVI were also excluded. Furthermore, patients who lacked essential baseline or follow-up data were excluded from the study. Only patients who underwent their first ablation procedure were included, and those who underwent redo ablation were excluded from the analysis.

Data extraction was performed by two investigators, who were initially blinded to the study hypotheses. Random samples of data were cross-checked to ensure accuracy. Patient data were de-identified and handled in compliance with relevant data protection regulations. This study adhered to the principles of the Declaration of Helsinki and was approved by the local ethics committee. Written informed consent was obtained from all participants prior to their inclusion in the study.

### Preprocedural management and ablation procedure

Preprocedural management followed established protocols 6, 11, 12. All patients underwent transesophageal echocardiography (TEE) and cardiac computed tomography angiography to assess pulmonary vein anatomy and rule out left atrial thrombus. TEE measurements were performed by an experienced cardiac sonographer. LVEF was measured using the modified biplane Simpson's method, and LAD was assessed via the left atrial left-right diameter. Antiarrhythmic drugs were discontinued for at least one week prior to the ablation procedure. The CHA_2_DS_2_-VASc and HAS-BLED scores were calculated for all patients to assess stroke and bleeding risks, and standardized oral anticoagulant therapy was initiated. For patients on warfarin, the international normalized ratio was maintained between 2.0 and 2.5 without interruption. For patients on novel oral anticoagulants (NOACs), medications were withheld 12 hours prior to ablation.

During the ablation procedure, heparin was administered immediately following interatrial septal puncture to maintain an activated clotting time between 300-400 seconds, with periodic monitoring every 15-30 minutes. All procedures were conducted under intravenous fentanyl sedation. In the RFA procedure, a CARTO magnetic mapping system (Biosense Webster, Inc.) or an Ensite three-dimensional mapping system (Saint Jude Medical, Inc.) was used to create a three-dimensional electroanatomic reconstruction of the left atrium (LA). PVI was performed under the guidance of three-dimensional electrical reconstruction of the LA and digital subtraction angiography. In the CBA procedure, following bilateral pulmonary vein angiography, a cryoballoon and the Achieve mapping catheters (Medtronic, Inc.) were sequentially advanced into the pulmonary veins for cryoablation. The Achieve mapping catheter was positioned at the ostium of each pulmonary vein to confirm PVI success. In the PFA procedure, which was performed under general anesthesia, a disposable cardiac PFA ablation catheter (Model number: PFA8D15LT) was connected to a dedicated PFA ablation generator. A three-dimensional model of the LA was constructed via a three-dimensional mapping system, and PVI was guided by this model (pulse amplitude: 1800 volts, pulse duration: 400 μs, interpulse interval: 450 milliseconds). Each pulmonary vein underwent 8-12 ablation cycles. All procedures were conducted by experienced electrophysiology teams.

### Postprocedural management and follow-up

Postprocedural management involves immediate echocardiographic assessment to rule out pericardial tamponade (PT) after ablation. Proton pump inhibitors were routinely prescribed for 4 weeks to prevent esophageal injury. Oral anticoagulation therapy was resumed on the day of the procedure and continued for 2 months. Stroke and bleeding risks were reassessed via the CHA_2_DS_2_-VASc and HAS-BLED scores to determine the need for continued anticoagulation therapy. Antiarrhythmic drugs were continued for 3 months post-ablation to prevent AF recurrence, with continuation thereafter on the basis of rhythm status.

Patients were followed up at 3, 6, and 12 months post-ablation, and every 6 months thereafter through outpatient visits, phone calls, or social media platforms. Follow-up assessments included evaluations of AF recurrence, quality of life scores [Kansas City Cardiomyopathy Questionnaire (KCCQ) score and Minnesota Living with Heart Failure Questionnaire (MLHFQ) score], NYHA functional classification, transthoracic echocardiography data (LAD and LVEF), and postprocedural complications. Standardized patient interviews were conducted by the same cardiologist. AF recurrence was defined as AF, atrial flutter or atrial tachycardia lasting more than 30 seconds, confirmed by 12-lead ECG or wearable devices, 3 months post-ablation. Postprocedural complications, including atherosclerotic cardiovascular disease (ASCVD) events, phrenic nerve palsy (PNP), pulmonary vein stenosis (PVS), PT, stroke or transient ischemic attack (TIA), and in-hospital death, were recorded.

### Statistical analysis

All continuous variables were assessed for normality of distribution using the Shapiro-Wilk test. Normally distributed continuous variables are presented as the means ± standard deviations, and between-group comparisons were performed via Student's t test. Nonnormally distributed continuous variables are presented as medians (25th, 75th percentiles), and between-group comparisons were performed via the Mann-Whitney U test. Categorical variables are expressed as proportions (%), with group comparisons performed via the χ^2^ test or Fisher's exact test. Paired-sample t tests were used to compare LAD and LVEF before and after ablation procedures, whereas KCCQ scores, MLHFQ scores, and NYHA functional classification were compared via paired-sample Wilcoxon signed-rank tests. In cases where significant differences (*P* < 0.05) were observed among multiple groups, post hoc tests were conducted for within-group comparisons. Kaplan-Meier (K-M) analysis was utilized to evaluate survival time distribution and to plot K-M curves, with the log-rank test used to compare significant differences between the curves. Cox proportional hazards regression was employed to calculate the hazard ratio (HR) for AF recurrence in patients with AF and HFpEF post-ablation, with *P* < 0.05 indicating statistical significance. All the statistical analyses were performed via IBM SPSS version 26 and GraphPad Prism version 9.4.0.

## Results

### Baseline characteristics

**Figure 1:**
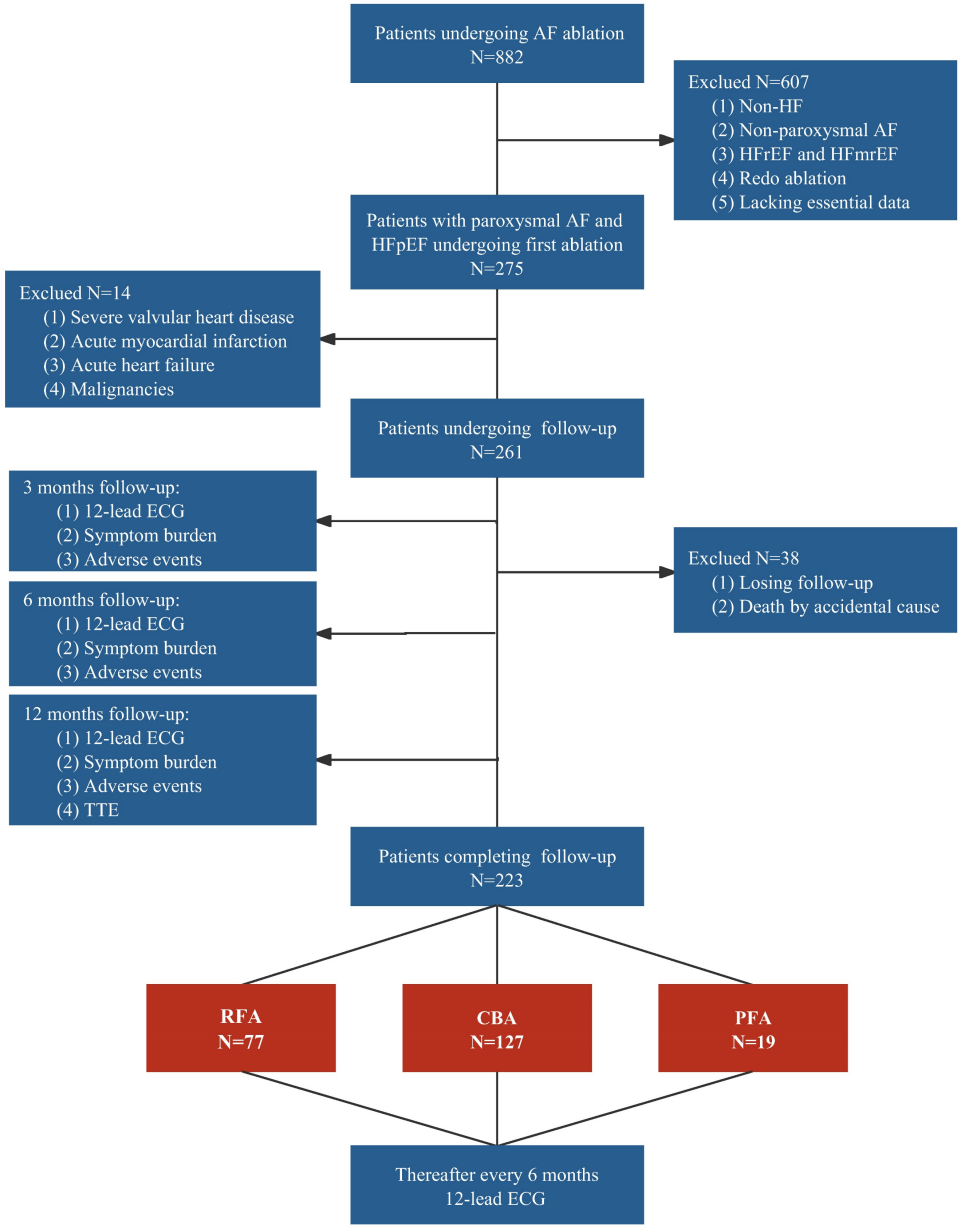
** Study flowchart.** AF, atrial fibrillation; CBA, cryoballoon ablation; ECG, electrocardiogram; HF, heart failure; HFmrEF, heart failure with mildly reduced ejection fraction; HFpEF, heart failure with preserved ejection fraction; HFrEF, heart failure with reduced ejection fraction; PFA, pulsed field ablation; RFA, radiofrequency ablation; TTE, transthoracic echocardiography.

We screened 882 patients with AF who underwent catheter ablation, including RFA, CBA, or PFA, at our center between 2017 and 2021. A total of 607 patients who lacked HF (n = 212), presented with non-paroxysmal AF (n = 165), had HFrEF or heart failure with mildly reduced ejection fraction (HFmrEF) (n = 187), underwent redo ablation (n = 40), or lacked essential baseline data (n = 3) were excluded. Among the remaining 275 patients, 14 patients were ineligible because of complications such as severe valvular heart disease (n = 8), acute myocardial infarction (n = 3), acute HF (n = 2), or malignancies (n = 1). Ultimately, 261 patients were included in the analysis. During the mean follow-up period of 11.2 ± 1.8 months, 38 patients were lost to follow-up, including one person who died from a non-cardiovascular-related accident, resulting in a dropout rate of 14.6%. The 223 patients who completed follow-up were categorized into the RFA group (34.5%), CBA group (57.0%), and PFA group (8.5%). The detailed process is illustrated in Figure 1.

The baseline characteristics of the 223 patients with paroxysmal non-valvular AF and HFpEF are described in Table 1. The median age was 64 (57-69) years, and 53.4% of the patients were male. The mean body mass index (BMI) was 24.2 ± 3.0 kg/m^2^, with no significant differences observed among the three groups (*P* = 0.658). Hypertension was the most common comorbidity, affecting 61.4% of the cohort. The median CHA_2_DS_2_-VASc and HAS-BLED scores were 2 (1-4) and 1 (0-2), respectively. The median KCCQ scores for the RFA, CBA, and PFA groups were 81.2 (75.0-81.2), 81.2 (56.7-81.2), and 75.0 (75.0-81.2), respectively. The median MLHFQ scores were 3 (3-3), 3 (3-26), and 3 (2-3) for the RFA, CBA, and PFA groups, respectively. No significant differences in the KCCQ or MLHFQ scores were observed between the groups (*P* = 0.298 and *P* = 0.392, respectively), nor were there differences in the NYHA functional classification (*P* = 0.081). A majority of patients (87.9%) were treated with NOACs, and 18.4% were on warfarin. The median levels of albumin (ALB) and NT-pro BNP were 42.1 (40.2-43.7) g/dL and 415.4 (377.1-649.1) pg/mL, respectively. Additionally, no significant intergroup differences were found in the baseline LAD or LVEF measurements (*P* = 0.926 and *P* = 0.264, respectively).

### Clinical endpoints and postprocedural complications

**Figure 2:**
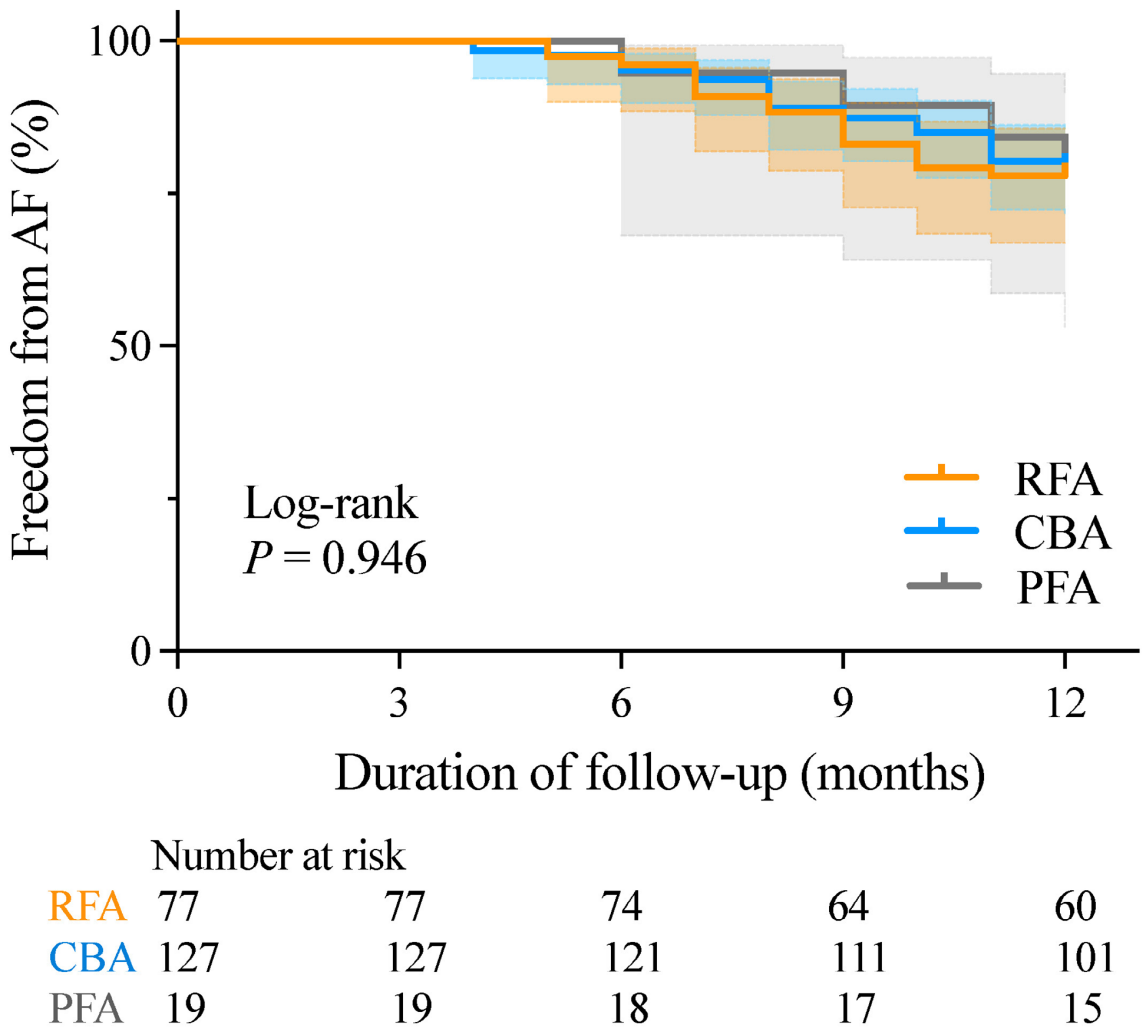
** K-M curves for freedom from AF recurrence.** AF, atrial fibrillation; CBA, cryoballoon ablation; K-M, Kaplan-Meier; PFA, pulsed field ablation; RFA, radiofrequency ablation.

There was no significant difference in AF recurrence rates between patients with paroxysmal non-valvular AF and those with HFpEF at 12 months post-RFA, CBA, or PFA (22.08% vs. 20.47% vs. 21.05%, respectively; *P* = 0.964). K-M survival analysis also revealed no significant differences in AF recurrence rates across the three groups during the mean follow-up period of 11.2 ± 1.8 months (log-rank *P* = 0.946) (Figure 2).

**Figure 3:**
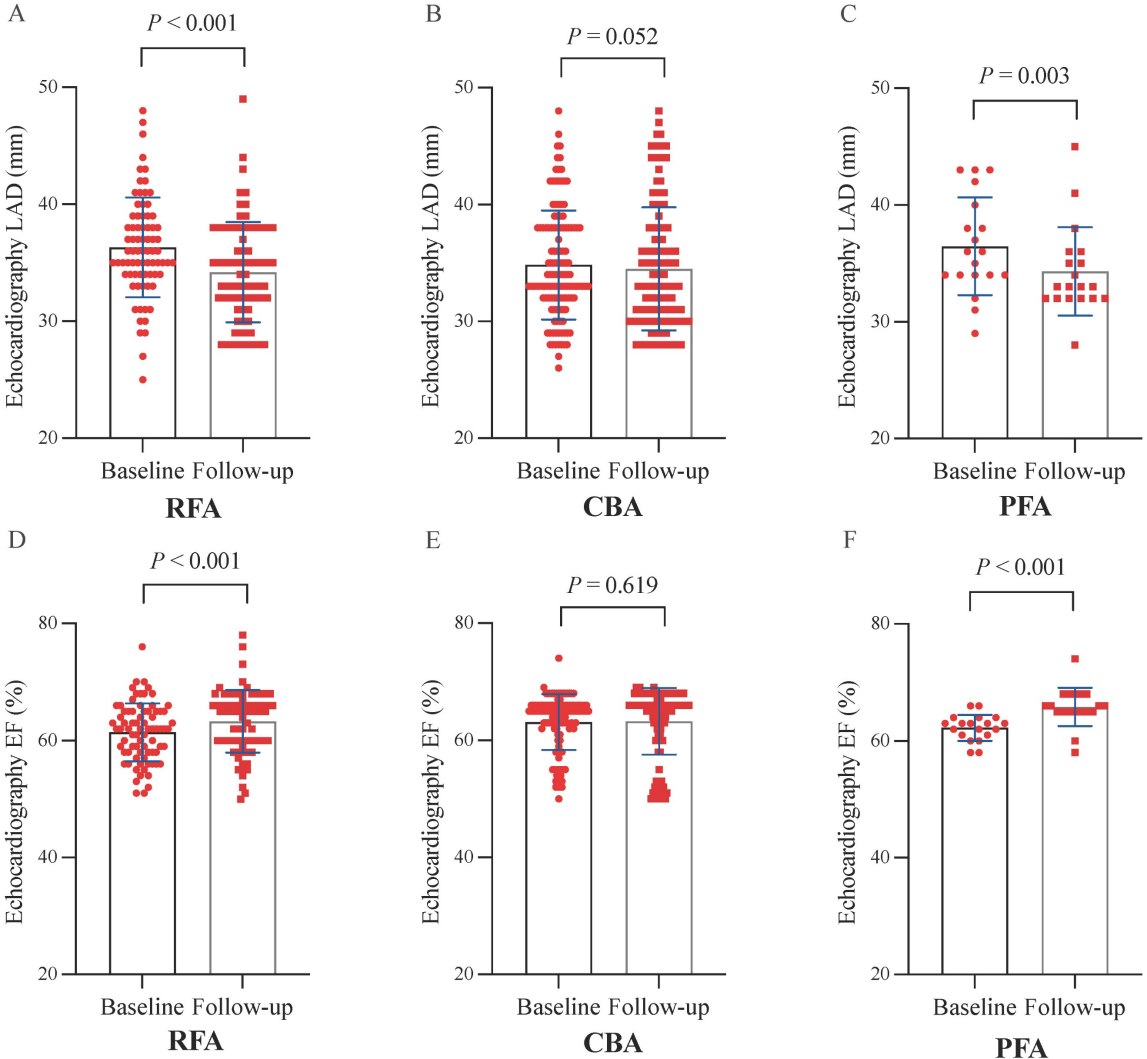
** Paired analyses of LAD and LVEF before and 12 months after the ablation procedure.** (A) Paired analyses of LAD in RFA. (B) Paired analyses of LAD in CBA. (C) Paired analyses of the LAD in the PFA. (D) Paired analyses of LVEF in the RFA group. (E) Paired analyses of LVEF in CBA. (F) Paired analyses of LVEF in PFA. CBA, cryoballoon ablation; LAD, left atrial diameter; LVEF, left ventricular ejection fraction; PFA, pulsed field ablation; RFA, radiofrequency ablation.

**Figure 4:**
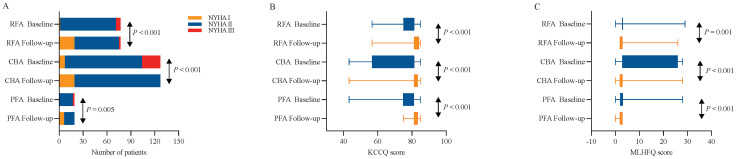
** Paired analyses of NYHA functional classification, KCCQ scores and MLHFQ scores before and 12 months after the ablation procedure.** (A) Paired analyses of the NYHA functional class in RFA, CBA and PFA. (B) Paired analyses of KCCQ scores in the RFA, CBA and PFA groups. (C) Paired analyses of the MLHFQ score in the RFA, CBA and PFA. CBA, cryoballoon ablation; KCCQ, Kansas City Cardiomyopathy Questionnaire; MLHFQ, Minnesota Living with Heart Failure Questionnaire; NYHA, New York Heart Association; PFA, pulsed field ablation; RFA, radiofrequency ablation.

**Figure 5:**
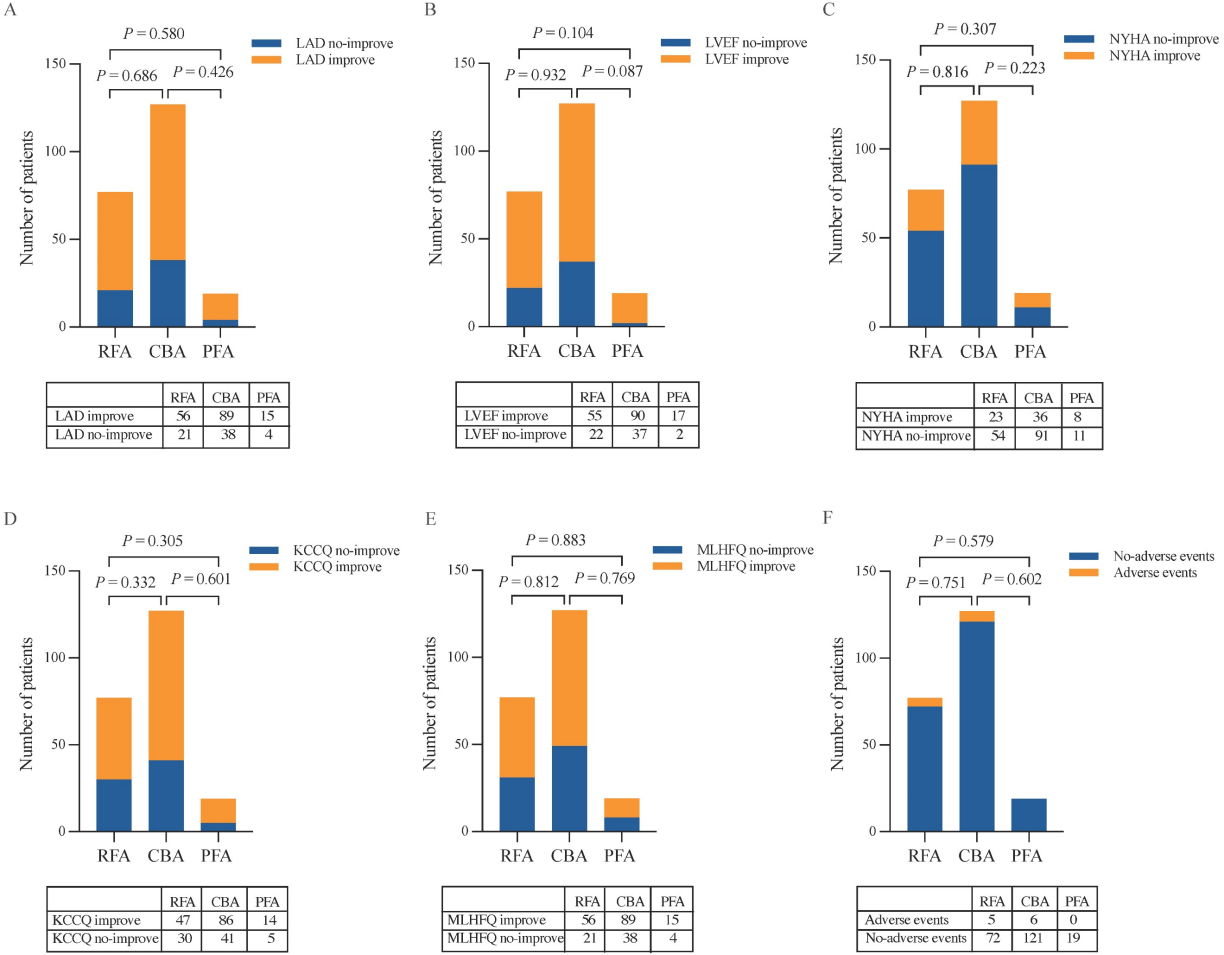
** Reassessment of LAD, LVEF, NYHA functional classification, KCCQ scores and MLHFQ scores 12 months after the ablation procedure and analysis of postprocedural complications.** (A) LAD. (B) LVEF. (C) NYHA functional classification. (D) KCCQ scores. (E) MLHFQ scores. (F) Postprocedural complications. CBA, cryoballoon ablation; KCCQ, Kansas City Cardiomyopathy Questionnaire; LAD, left atrial diameter; LVEF, left ventricular ejection fraction; MLHFQ, Minnesota Living with Heart Failure Questionnaire; NYHA, New York Heart Association; PFA, pulsed field ablation; RFA, radiofrequency ablation.

At 12 months post-ablation, significant reductions in the LAD were observed in the RFA group (34.61 ± 4.23 mm vs. 36.32 ± 4.26 mm, *P* < 0.001) and PFA group (34.32 ± 3.79 mm vs. 36.47 ± 4.18 mm, *P* = 0.003) compared with the baseline values (Figure 3A, C). Furthermore, no significant difference was found in the percentage change in the LAD between the two groups (*P* = 0.580) (Figure 5A). In contrast, no significant change in the LAD was observed in the CBA group post-ablation (34.50 ± 5.27 mm vs. 36.46 ± 4.53 mm, *P* = 0.052) (Figure 3B). Both the RFA group (63.58 ± 5.29% vs. 61.40 ± 4.90%, *P* < 0.001) and the PFA group (65.84 ± 3.25% vs. 62.30 ± 2.20%, *P* < 0.001) exhibited significant increases in LVEF post-ablation compared with baseline (Figure 3D, F), with no significant difference in the percentage increase in LVEF between the two groups (*P* = 0.104) (Figure 5B). However, there was no significant change in LVEF before and after ablation in the CBA group (63.25 ± 5.66% vs. 61.00 ± 4.40%, *P* = 0.619) (Figure 3E). Additionally, patients in the RFA (*P* < 0.001), CBA (*P* < 0.001), and PFA (*P* = 0.005) groups demonstrated significant improvements in the NYHA functional classification post-ablation, relative to baseline (Figure 4A), although no significant differences were observed in the percentage of improvement between the groups (*P* = 0.816; *P* = 0.223; *P* = 0.307) (Figure 5C). Significant improvements in patient quality of life scores were also observed across all groups, with similar improvements in KCCQ scores and MLHFQ scores (Figure 4B, C and Figure 5D, E). Detailed follow-up data are provided in Supplementary Table 1.

During the follow-up period, there were no significant differences in the incidence of postprocedural complications among the three groups (*P* = 0.751; *P* = 0.602; *P* = 0.579, respectively) (Figure 5F). The detailed postprocedural complication events are provided in Table 2.

### Predictors of AF recurrence post-ablation

Univariate Cox regression analysis identified ALB and NT-pro BNP as potential predictors of AF recurrence (Supplementary Table 2). After adjusting for confounding variables, multivariate Cox regression analysis demonstrated that both ALB and NT-pro BNP were independent predictors of AF recurrence, with HR values of 0.612 [95% confidence interval (CI) 0.512-0.732, *P* < 0.001] and 1.003 (95% CI 1.002-1.004, *P* < 0.001), respectively (Model 3, Table 3).

## Discussion

This study revealed the following key findings: (1) Both RFA and PFA effectively maintained sinus rhythm, reversed atrial remodeling, and increased LVEF in patients with paroxysmal non-valvular AF and HFpEF post-ablation, whereas CBA only sustained sinus rhythm without reversing atrial remodeling or increasing LVEF. (2) RFA, CBA, and PFA improved patients' quality of life scores and NYHA functional classification post-ablation. (3) No significant differences were observed in the incidence of postprocedural complications among the three groups, with rare severe complications. (4) ALB and NT-pro BNP were identified as independent predictors of AF recurrence in patients with paroxysmal non-valvular AF and HFpEF.

AF and HFpEF are frequently coexisting conditions that may exacerbate each other through various mechanisms 13. The incidence of HFpEF in patients with AF is approximately five times greater than that in patients without AF, and more than 30% of patients with HFpEF experience concomitant AF 14, 15. In patients with acutely decompensated HFpEF, AF can be observed in up to 69% of cases 16. Furthermore, the presence of AF in patients with HFpEF significantly increases the risk of both all-cause mortality and stroke, particularly when AF is persistent 17. Because of the intricate pathophysiological mechanisms involved in HFpEF, the clinical benefits of guideline-directed medical therapy are less pronounced than those in patients with HFrEF. Early rhythm control strategies, particularly catheter ablation, have emerged as promising treatment approaches. The EAST-AFNET 4 trial (NCT01288352) underscores the substantial benefits of early rhythm control strategies in both symptomatic and asymptomatic AF patients 18. This approach was used in patients with HF (n = 798), a majority of whom had HFpEF (56%). Proactive implementation of early rhythm control is pivotal for patients with AF and HFpEF.

Previous studies have demonstrated the efficacy of RFA in patients with AF and HFpEF 11, 19-21. The STALL AF-HFpEF trial indicated that RFA could reduce pulmonary capillary wedge pressure and improve HFpEF symptoms in patients with AF and HFpEF 22. Moreover, a meta-analysis comprising seven trials (n = 1696) indicated that RFA effectively preserves sinus rhythm in patients with HFpEF, with significant improvements in reducing rehospitalization compared with medical therapy alone 23. Recently, a randomized, prospective, single-blinded, controlled trial involving 31 patients with AF and HFpEF (16 for RFA vs. 15 for medical therapy) revealed that RFA improved invasive exercise-related hemodynamic parameters, exercise capacity, and quality of life 19. In this study, RFA was effective in maintaining sinus rhythm post-ablation, reversing left atrial remodeling, and increasing the LVEF. Additionally, it improved quality of life scores and NYHA functional classification. These findings align with those of previous studies, confirming the benefits of RFA for patients with paroxysmal non-valvular AF and HFpEF. Furthermore, the incidence of postprocedural complications in the RFA group was low, supporting its safety and efficacy as an ablation strategy for this patient population.

CBAs offer potential advantages for patients with HF, as they eliminate the additional fluid burden induced by irrigation catheters and reduce the incidence of complications 24. A single-center retrospective study indicated that CBA could reverse remodeling, improve symptoms, and reduce HFpEF-related hospitalizations 6. An analysis by Chen *et al.* 25 involving 471 patients with AF, including 101 with HFpEF, who underwent CBA revealed significant benefits for HFpEF patients, with high maintenance of sinus rhythm and notable improvement in HF. Additionally, data from the Cryo AF Global Registry (trial code NCT02752737), encompassing 318 patients with AF and HF (81.6% of whom had HFpEF), indicated that CBA could improve AF-related symptoms and reduce the reliance on antiarrhythmic drugs 26. Despite the initial efficacy observed in patients with AF and HFpEF, current research on the role of CBA in treating patients with AF and HFpEF is limited, and evidence from large-scale randomized controlled trials is lacking. Notably, 102 patients with HFpEF who underwent CBA were more susceptible to AF recurrence (57% vs. 23%, *P* = 0.003), repeat AF ablation (39% vs. 14%, *P* = 0.01), and AF-related rehospitalization (26% vs. 7%, *P* = 0.016) than patients without HFpEF 7. Even in patients with HFpEF who achieve sinus rhythm following ablation, persistent HF symptoms and elevated cardiac biomarkers raise questions about the overall efficacy of CBA in managing AF combined with HFpEF. In this study, CBA did not significantly reverse left atrial remodeling or improve LVEF, but it effectively enhanced the quality of life scores and NYHA functional classification. Furthermore, the incidence of postprocedural complications in the CBA group was low, making it a relatively safe ablation strategy. Consequently, the efficacy of CBA in patients with paroxysmal non-valvular AF and HFpEF remains contentious. Large-scale randomized controlled trials are needed to further assess the efficacy of CBA in this population.

PFA is a nonthermal method of tissue ablation that uses high-amplitude pulsed electrical fields to create irreversible electroporation, which does not cause significant protein denaturation or damage to tissue scaffolding 27. Cardiomyocytes have one of the lowest threshold values for any tissue. PFA uses ultrarapid electrical pulses to ablate target cardiomyocytes while sparing surrounding tissue preferentially 28. PFA is a promising strategy for AF ablation. The PULSED AF Pivotal trial (trial code NCT04198701) indicated that PFA was effective in 66.2% (95% CI 57.9-73.2) of patients with paroxysmal AF and 55.1% (95% CI 46.7-62.7) of patients with persistent AF at 12 months, with a low rate of adverse events (0.7%) 12. Data from the MANIFEST-PF Registry also revealed that PFA was clinically effective in 78% of patients with AF 29. Currently, studies on the efficacy and safety of PFA in treating patients with AF and HFpEF are lacking. To our knowledge, this is the first study to systematically analyze the efficacy and safety of PFA in patients with paroxysmal non-valvular AF and HFpEF. Our findings indicated that PFA can effectively maintain sinus rhythm, reverse left atrial structural remodeling, increase LVEF, and improve both quality of life scores and NYHA functional classification. Additionally, the postprocedural complication rate of PFA is relatively low, positioning PFA as a promising new ablation technique for AF in patients with HFpEF.

Our multivariate Cox regression analyses revealed that the serum ALB and NT-pro BNP levels were independent predictors of AF recurrence in patients with HFpEF. In general, the ALB concentration and NT-pro BNP level reflect the patient's nutritional status and the severity of HF 30, 31. Existing evidence indicates that the ALB concentration is associated with myocardial fibrosis, adverse pulsatile aortic hemodynamics, and prognosis in patients with HFpEF. The ALB concentration has been identified as a strong predictor of death or hospitalization in patients with HF 32. Monitoring the ALB concentration may enhance risk stratification in patients with HFpEF. Biomarkers play crucial roles in assessing the individual risk of HF. Abundant evidence suggests an association between lower levels of NT-pro BNP and mild HF 33. At present, there are no biomarkers that are superior to natriuretic peptides in terms of the diagnosis and prognosis of HFpEF 34. A single value of NT-pro BNP >5000 pg/mL predicts a worse outcome in hospitalized patients with HFrEF. In stable outpatients with HFrEF, an NT-pro BNP > 1000 pg/mL predicts a poorer prognosis. Notably, NT-pro BNP levels provide similar prognostic information in patients with HFpEF and those with HFrEF 35. A comprehensive evaluation of the prognostic relevance of biomarkers such as the ALB concentration and NT-pro BNP level is pivotal for evaluating the overall risk of AF recurrence in patients with HFpEF. Further prospective studies are positioning PFA as a promising new ablation technique for AF in patients with HFpEF.

### Limitations

This study has several limitations: (1) This was a retrospective observational study, and the sample size may be insufficient to draw definitive conclusions. Therefore, large-scale randomized controlled trials are essential to validate the replicability and generalizability of these findings. (2) The absence of standardized arrhythmia detection strategies (e.g., implantable loop records) may have led to an underestimation of AF recurrence. (3) This study focused primarily on patients with paroxysmal non-valvular AF and HFpEF, excluding those with persistent AF and HFpEF. Consequently, our conclusions are specific to this subgroup and cannot be generalized to the broader population of patients with AF and HFpEF. Additional prospective studies are needed to assess the efficacy and safety of CBA and PFA in patients with persistent AF and HFpEF. (4) There is a lack of consensus on the precise diagnostic criteria for HFpEF. Although the H2FPEF score was utilized to assist in the diagnosis of HFpEF, the retrospective diagnosis of HFpEF still presents certain challenges, potentially impacting the study outcomes. (5) The study findings revealed that there were no postprocedural complications in the PFA group during the 12 months after the ablation procedure. However, owing to the relatively small sample size in the PFA group and the absence of long-term follow-up, future prospective studies are needed to confirm the efficacy and safety of PFA in patients with AF and HFpEF.

## Conclusion

In summary, our study compared the efficacy and safety of RFA, CBA, and PFA in patients with paroxysmal non-valvular AF and HFpEF. Both RFA and PFA were effective in maintaining sinus rhythm post-ablation, reversing left atrial remodeling, and increasing LVEF. While CBA significantly improved post-ablation quality of life scores and NYHA functional classification, it did not reverse left atrial structural remodeling or increase LVEF. The incidence of postprocedural complications was similar across all three groups, indicating good safety profiles. Additionally, the ALB concentration and NT-pro BNP level emerged as independent predictors of AF recurrence, providing important insights for risk stratification and potential early intervention strategies in patients with paroxysmal non-valvular AF and HFpEF. Large-scale prospective studies in the future could validate these findings and further refine treatment strategies for patients with AF and HFpEF.

## Supplementary Material

Supplementary tables.

## Figures and Tables

**Table 1:** Demographic and baseline characteristics of the study patients

Variables	All	RFA	CBA	PFA	*P* value
	N = 223	n = 77	n = 127	n = 19	
Demographics					
Age (y)	64 (57-69)	65 (57-69)	64 (56-71)	61 (59-66)	0.618
Male (%)	119 [53.4]	41 [53.2]	69 [54.3]	9 [47.4]	0.851
SBP (mmHg)	134.5 ± 18.3	134.9 ± 19.7	134.2 ± 17.3	134.9 ± 20.6	0.957
DBP (mmHg)	79.6 ± 10.8	79.7 ± 12.5	79.2 ± 10.0	81.8 ± 8.5	0.567
Heart rate (beats/min)	72.6 ± 11.9	70.7 ± 10.7	74.1 ± 13.0	69.6 ± 7.8	0.164
BMI (kg/m^2^)	24.2 ± 3.0	24.2 ± 2.2	24.0 ± 3.2	25.2 ± 4.3	0.658
CHA_2_DS_2_-VASc score	2 (1-4)	2 (1-3)	2 (1-4)	3 (1-4)	0.524
HAS-BLED score	1 (0-2)	1 (0-2)	1 (0-2)	1 (1-2)	0.946
Comorbidities					
Hypertension (n, %)	137 [61.4]	44 [57.1]	80 [63.0]	13 [68.4]	0.571
Diabetes (n, %)	33 [14.8]	9 [11.7]	20 [15.7]	4 [21.1]	0.530
CHD (n, %)	65 [29.1]	24 [31.2]	37 [29.1]	4 [21.1]	0.686
Stroke (n, %)	37 [16.6]	10 [13.0]	22 [17.3]	5 [26.3]	0.355
COPD (n, %)	8 [3.6]	4 [5.2]	4 [3.1]	0 [0]	0.509
OSAHS (n, %)	6 [2.7]	1 [1.3]	3 [2.4]	2 [22.2]	0.079
QOL measurements					
KCCQ score	81.2 (75.0-81.2)	81.2 (75.0-81.2)	81.2 (56.7-81.2)	75.0 (75.0-81.2)	0.298
MLHFQ score	3 (3-3)	3 (3-3)	3 (3-26)	3 (2-3)	0.392
NYHA class (n, %)					0.081
I	8 [3.6]	1 [1.3]	7 [5.5]	0 [0]	
II	184 [82.5]	70 [90.9]	97 [76.4]	17 [89.5]	
III	31 [13.9]	6 [7.8]	23 [18.1]	2 [10.5]	
Medical treatment					
Aspirin (n, %)	36 [16.1]	12 [15.6]	23 [18.1]	1 [5.3]	0.360
Clopidogrel (n, %)	25 [11.2]	8 [10.4]	16 [12.6]	1 [5.3]	0.615
Warfarin (n, %)	41 [18.4]	15 [19.5]	26 [20.5]	0 [0]	0.095
NOACs (n, %)	196 [87.9]	64 [83.1]	113 [89.0]	19 [100.0]	0.110
β blocker (n, %)	126 [56.5]	39 [50.6]	77 [60.6]	10 [52.6]	0.355
Statin (n, %)	145 [65]	48 [62.3]	83 [65.4]	14 [73.7]	0.645
Laboratory data					
eGFR (mL/min/1.73m^2^)	85.9 (74.8-95.6)	89.3 (73.0-96.5)	85.8 (75.3-95.6)	82.1 (71.8-95.6)	0.648
UA (umol/L)	330.1 (279.8-378.8)	323.5 (291.5-401.8)	333.0 (277.0-379.2)	313.0 (265.0-369.0)	0.693
TG	1.3 (0.9-1.9)	1.3 (1.0-1.9)	1.3 (0.9-1.8)	1.3 (0.9-2.1)	0.796
ALB (g/dL)	42.1 (40.2-43.7)	41.9 (40.0-43.8)	42.3 (40.4-43.7)	42.1 (39.9-43.6)	0.774
NT-pro BNP (pg/ml)	415.4 (377.1-649.1)	426.9 (389.7-677.5)	417.0 (378.5-735.6)	402.0 (388.0-412.6)	0.144
TEE					
LAD (mm)	36.4 ± 4.4	36.3 ± 4.3	36.5 ± 4.5	36.5 ± 4.2	0.926
LVEF (%)	61.3 ± 4.5	61.4 ± 4.9	61.0 ± 4.4	62.3 ± 2.2	0.264

The values are presented as the means ± standard deviations, medians (interquartile ranges) or n [%]. A *P* values < 0.05 indicated a significant difference. ALB, albumin; BMI, body mass index; CBA, cryoballoon ablation; CHA_2_DS_2_-VASc, congestive heart failure, hypertension, age ≥75 years, diabetes mellitus, stroke, vascular disease, age 65-74 years, sex category; CHD, coronary heart disease; COPD, chronic obstructive pulmonary disease; DBP, diastolic blood pressure; eGFR, estimated glomerular filtration rate; HAS-BLED, hypertension, abnormal renal/liver function, stroke, bleeding history or predisposition, labile international normalized ratio, elderly, drugs/alcohol concomitantly; KCCQ, Kansas City Cardiomyopathy Questionnaire; LAD, left atrial diameter; LVEF, left ventricular ejection fraction; MLHFQ, Minnesota Living with Heart Failure Questionnaire; NOACs, novel oral anticoagulants; NT-pro BNP, N-terminal pro-B-type natriuretic peptide; NYHA, New York Heart Association; OSAHS, obstructive sleep apnea-hypopnea syndrome; PFA, pulsed field ablation; QOL, quality of life; RFA, radiofrequency ablation; SBP, systolic blood pressure; TEE, transesophageal echocardiography; TG, triglyceride; UA, uric acid.

**Table 2:** The number of postprocedural complications

	Total	ASCVD	Stroke/TIA	PVS	PNP	PT	In-hospital death
RFA	5	2	2	1	0	0	0
CBA	6	1	3	1	1	0	0
PFA	0	0	0	0	0	0	0

ASCVD, atherosclerotic cardiovascular disease; CBA, cryoballoon ablation; PFA, pulsed field ablation; PNP, phrenic nerve palsy; PT, pericardial tamponade; PVS, pulmonary vein stenosis; RFA, radiofrequency ablation; TIA, transient ischemic attack.

**Table 3:** Cox regressions for identifying predictors of AF recurrence 12 months post-ablation

Variables		Unadjusted				Model 1				Model 2				Model 3		
		HR	95% CI	*P* value		HR	95% CI	*P* value		HR	95% CI	*P* value		HR	95% CI	*P* value
Age		0.991	0.961-1.022	0.571												
Sex		0.666	0.370-1.200	0.176												
BMI		0.963	0.873-1.061	0.446												
CHA_2_DS_2_-VASc		0.945	0.806-1.107	0.482		1.005	0.814-1.242	0.962								
NYHA class		1.823	0.942-3.528	0.075		2.343	1.147-4.786	0.019								
KCCQ score		0.985	0.966-1.005	0.146		0.980	0.960-1.001	0.059								
MLHFQ score		1.025	0.999-1.051	0.056		1.029	1.003-1.057	0.031								
Hypertension		0.775	0.436-1.378	0.386		0.786	0.434-1.422	0.426								
Diabetes		0.492	0.177-1.371	0.175		0.528	0.187-1.486	0.226								
CHD		1.138	0.616-2.101	0.680		1.320	0.683-2.553	0.409								
Stroke		1.067	0.499-2.283	0.868		1.103	0.505-2.410	0.806								
COPD		1.308	0.317-5.392	0.711		1.132	0.266-4.820	0.866								
eGFR		1.006	0.989-1.024	0.501		1.005	0.985-1.026	0.624		1.001	0.980-1.022	0.930				
UA		1.000	0.997-1.004	0.792		1.000	0.996-1.003	0.897		1.000	0.996-1.004	0.990				
TG		0.919	0.683-1.235	0.574		0.918	0.678-1.243	0.580		0.939	0.697-1.265	0.679				
LAD		1.024	0.960-1.092	0.478		1.040	0.969-1.117	0.277		1.038	0.967-1.116	0.303				
LVEF		1.051	0.982-1.125	0.150		1.053	0.982-1.129	0.151		1.067	0.991-1.149	0.088				
ALB		0.637	0.541-0.749	< 0.001		0.629	0.533-0.742	< 0.001		0.634	0.535-0.752	< 0.001		0.612	0.512-0.732	< 0.001
NT-pro BNP		1.002	1.002-1.003	< 0.001		1.002	1.002-1.003	< 0.001		1.003	1.002-1.003	< 0.001		1.003	1.002-1.004	< 0.001

A *P* value < 0.05 indicated a significant difference. AF, atrial fibrillation; ALB, serum albumin; BMI, body mass index; CHA_2_DS_2_-VASc, congestive heart failure, hypertension, age ≥75 years, diabetes mellitus, stroke, vascular disease, age 65-74 years, sex category; CHD, coronary heart disease; CI, confidence interval; COPD, chronic obstructive pulmonary disease; eGFR, estimated glomerular filtration rate; HR, hazard ratio; KCCQ, Kansas City Cardiomyopathy Questionnaire; LAD, left atrial diameter; LVEF, left ventricular ejection fraction; MLHFQ, Minnesota Living with Heart Failure Questionnaire; NT-pro BNP, N-terminal pro-B-type natriuretic peptide; NYHA, New York Heart Association; TG, triglyceride; UA, uric acid. Model 1 was adjusted for age, sex, and BMI. Model 2 was adjusted for age, sex, BMI, CHA_2_DS_2_-VASc score, NYHA functional classification, KCCQ score, MLHFQ score, hypertension, diabetes, CHD, stroke, and COPD. Model 3 was adjusted for age, sex, BMI, CHA_2_DS_2_-VASc score, NYHA functional classification, KCCQ score, MLHFQ score, hypertension, diabetes, CHD, stroke, COPD, eGFR, UA, TG, LAD, and LVEF.

## Abbreviations

AF: atrial fibrillation
ALB: albumin
ASCVD: atherosclerotic cardiovascular disease
BMI: body mass index
CBA: cryoballoon ablation
CHD: coronary heart disease
CI: confidence interval
COPD: chronic obstructive pulmonary disease
DBP: diastolic blood pressure
ECG: electrocardiogram
eGFR: estimated glomerular filtration rate
HF: heart failure
HR: hazard ratio
HFmrEF: heart failure with mildly reduced ejection fraction
HFpEF: heart failure with preserved ejection fraction
HFrEF: heart failure with reduced ejection fraction
KCCQ: Kansas City Cardiomyopathy Questionnaire
K-M: Kaplan-Meier
LA: left atrium
LAD: left atrial diameter
LVEF: left ventricular ejection fraction
MLHFQ: Minnesota Living with Heart Failure Questionnaire
NOACs: novel oral anticoagulants
NT-pro BNP: N-terminal pro-B-type natriuretic peptide
NYHA: New York Heart Association
OSAHS: obstructive sleep apnea-hypopnea syndrome
PFA: pulsed field ablation
PNP: phrenic nerve palsy
PT: pericardial tamponade
PVI: pulmonary vein isolation
PVS: pulmonary vein stenosis
RFA: radiofrequency ablation
SBP: systolic blood pressure
TEE: transesophageal echocardiography
TG: triglyceride
TIA: transient ischemic attack
UA: uric acid
