# Supplementary material for: Comparison of Catheter Ablation in Patients with Paroxysmal Non-valvular Atrial Fibrillation and Heart Failure with Preserved Ejection Fraction
Source: Int J Med Sci. 2025 Jan 1;22(2):371–82. doi: 10.7150/ijms.103170 (PMC11704700; doi:10.7150/ijms.103170)
Supplement: Supplementary file 1 — Supplementary tables. [file ijmsv22p0371s1.pdf]

Supplementary Table 1. Detailed follow-up data.

Variable	All<br>N = 223	RFA<br>n = 77	CBA<br>n = 127	PFA<br>n = 19	*P* value
AF recurrence	47	17	26	4	0.964
KCCQ score	83.30 (81.20–83.30)	83.30 (81.20–84.15)	83.30 (81.20–83.30)	83.30 (81.20–83.30)	0.608
MLHFQ score	2 (2–3)	2 (2–3)	2 (2–3)	2 (2–3)	0.436
NYHA functional class					0.164
I	44 [19.7]	19 [24.7]	19 [15.0]	6 [31.6]	
II	177 [79.4]	56 [72.7]	108 [85.0]	13 [68.4]	
III	2 [0.9]	2 [2.6]	0 [0.0]	0 [0.0]	
LAD	34.52 ± 4.80	34.61 ± 4.23	34.50 ± 5.27	34.32 ± 3.79	0.631
LVEF	63.59 ± 5.40	63.58 ± 5.29	63.25 ± 5.66	65.84 ± 3.25	0.348

The values are presented as the means ± standard deviations, medians (interquartile ranges) or n [%]. A *P* value < 0.05 indicated a significant difference. AF, atrial fibrillation; CBA, cryoballoon ablation; KCCQ, Kansas City Cardiomyopathy Questionnaire; LAD, left atrial diameter; LVEF, left ventricular ejection fraction; MLHFQ, Minnesota Living with Heart Failure Questionnaire; NYHA, New York Heart Association; PFA, pulsed field ablation; RFA, radiofrequency ablation.

Supplementary Table 2. Univariate Cox regressions were conducted to identify predictors of AF recurrence 12 months post-ablation.

Variables	Unadjusted		
	HR	95% CI	*P* value
Age	0.991	0.961–1.022	0.571
Sex	0.666	0.370–1.200	0.176
BMI	0.963	0.873–1.061	0.446
CHA_2DS_2-VASc	0.945	0.806–1.107	0.482
NYHA class	1.823	0.942–3.528	0.075
KCCQ score	0.985	0.966–1.005	0.146
MLHFQ score	1.025	0.999–1.051	0.056
Hypertension	0.775	0.436–1.378	0.386
Diabetes	0.492	0.177–1.371	0.175
CHD	1.138	0.616–2.101	0.680
Stroke	1.067	0.499–2.283	0.868
COPD	1.308	0.317–5.392	0.711
eGFR	1.006	0.989–1.024	0.501
UA	1.000	0.997–1.004	0.792
TG	0.919	0.683–1.235	0.574
ALB	0.637	0.541–0.749	**< 0.001**
NT-pro BNP	1.002	1.002–1.003	**< 0.001**
LAD	1.024	0.960–1.092	0.478
LVEF	1.051	0.982–1.125	0.150

Boldface denotes significant *P* values. A *P* value < 0.05 indicated a significant difference. AF, atrial fibrillation; ALB, serum albumin; BMI, body mass index; CHA_2DS_2-VASc, congestive heart failure, hypertension, age ≥75 years, diabetes mellitus, stroke, vascular disease, age 65–74 years, sex category; CHD, coronary heart disease; CI, confidence interval; COPD, chronic obstructive pulmonary disease; eGFR, estimated glomerular filtration rate; HR, hazard ratio; KCCQ, Kansas City Cardiomyopathy Questionnaire; LAD, left atrial diameter; LVEF, left ventricular ejection fraction; MLHFQ, Minnesota Living with Heart Failure Questionnaire; NT-pro BNP, N-terminal pro-B-type natriuretic peptide; NYHA, New York Heart Association; TG, triglyceride; UA, uric acid.
